# The Economic, Medical and Psychosocial Consequences of Whole Genome Sequencing for the Genetic Diagnosis of Patients With Intellectual Disability: The DEFIDIAG Study Protocol

**DOI:** 10.3389/fgene.2022.852472

**Published:** 2022-04-04

**Authors:** Catherine Lejeune, Charley Robert-Viard, Nicolas Meunier-Beillard, Myriam Alice Borel, Léna Gourvès, Stéphanie Staraci, Anne-Laure Soilly, Francis Guillemin, Valerie Seror, Hamza Achit, Marion Bouctot, Marie-Laure Asensio, Anne-Sophie Briffaut, Christelle Delmas, Ange-Line Bruel, Alexia Benoit, Alban Simon, Bénédicte Gerard, Hamza Hadj Abdallah, Stanislas Lyonnet, Laurence Faivre, Christel Thauvin-Robinet, Sylvie Odent, Delphine Heron, Damien Sanlaville, Thierry Frebourg, Jean Muller, Yannis Duffourd, Anne Boland, Jean-François Deleuze, Hélène Espérou, Christine Binquet, Hélène Dollfus

**Affiliations:** ^1^ CHU Dijon Bourgogne, Inserm, Université de Bourgogne, CIC 1432, Module Épidémiologie Clinique, Dijon, France; ^2^ Inserm, Université Bourgogne-Franche-Comté, UMR 1231, EPICAD, Dijon, France; ^3^ CHU Dijon Bourgogne, Délégation à la Recherche Clinique et à l’Innovation, USMR, Dijon, France; ^4^ Observatoire Régional de Santé Bourgogne Franche-Comté, Dijon, France; ^5^ CHU Dijon Bourgogne, Direction de la Recherche Clinique, Dijon, France; ^6^ Unité Fonctionnelle de Génétique Médicale et Centre de Référence « Déficiences Intellectuelles de Causes Rares », APHP Sorbonne Université, Groupe Hospitalier Pitié-Salpêtrière et Hôpital Trousseau, Paris, France; ^7^ CIC1433-Epidémiologie Clinique, Centre Hospitalier Régional et Universitaire, Inserm, Université de Lorraine, Nancy, France; ^8^ Aix Marseille Univ, IRD, APHM, SSA, VITROME, IHU-Méditerranée Infection, Marseille, France; ^9^ Inserm, Pôle de Recherche Clinique, Paris, France; ^10^ CHU Dijon Bourgogne, Fédération Hospitalo-Universitaire Médecine Translationnelle et Anomalies du Dévelopment (TRANSLAD), Inserm, Université Bourgogne-Franche-Comté, UMR1231, Équipe GAD, Dijon, France; ^11^ Laboratoires de Diagnostic Génétique, Institut de Génétique Médicale d’Alsace (IGMA), Hôpitaux Universitaires de Strasbourg, Strasbourg, France; ^12^ Inserm UMRS_1112, Institut de Génétique Médicale d’Alsace, Université de Strasbourg, France et Service de Génétique Médicale Hôpitaux Universitaires de Strasbourg, Strasbourg, France; ^13^ Inserm, IHU *Imagine*—Institut des Maladies Génétiques, Université Paris Cité, Paris, France; ^14^ Fédération de Génétique et Médecine Génomique, Hôpital Necker-Enfants Malades, GHU APHP. Centre-Université Paris Cité, Paris, France; ^15^ Service de Génétique Clinique, Centre de Référence Anomalies du Dévelopment CLAD- Ouest, CNRS, IGDR UMR6290 (Institut de Génétique et Dévelopment de Rennes), ERN ITHACA, Université de Rennes, Rennes, France; ^16^ Hospices Civils de Lyon, GHE, Service de Génétique, Université Claude Bernard Lyon 1, Lyon, France; ^17^ CHU de Rouen, Service de Génétique, Rouen, France; ^18^ Inserm, UMR1245, Centre de Génomique et de Médecine Personnalisée, Université de Normandie, Rouen, France; ^19^ Unité Fonctionnelle de Bioinformatique Médicale Appliquée au Diagnostic (UF7363), Hôpitaux Universitaires de Strasbourg, Strasbourg, France; ^20^ CEA, Centre National de Recherche en Génomique Humaine (CNRGH), Université Paris-Saclay, Evry, France

**Keywords:** intellectual disability, genome sequencing, cost-effectiveness, qualitative study, micro-costing

## Abstract

**Introduction:** Like other countries, France has invested in a national medical genomics program. Among the four pilot research studies, the DEFIDIAG project focuses on the use of whole genome sequencing (WGS) for patients with intellectual disability (ID), a neurodevelopmental condition affecting 1–3% of the general population but due to a plethora of genes. However, the access to genomic analyses has many potential individual and societal issues in addition to the technical challenges. In order to help decision-makers optimally introduce genomic testing in France, there is a need to identify the socio-economic obstacles and leverages associated with the implementation of WGS.

**Methods and Analysis:** This humanities and social sciences analysis is part of the DEFIDIAG study. The main goal of DEFIDIAG is to compare the percentage of causal genetic diagnoses obtained by trio WGS (including the patient and both parents) (WGS_T_) to the percentage obtained using the minimal reference strategy currently used in France (Fragile-X testing, chromosomal microarray analysis, and gene panel strategy including 44 ID genes) for patients with ID having their first clinical genetics consultation. Additionally, four complementary studies will be conducted. First, a cost-effectiveness analysis will be undertaken in a subsample of 196 patients consulting for the first time for a genetic evaluation; in a blinded fashion, WGS_T_ and solo (index case, only) genomic analysis (WGS_S_) will be compared to the reference strategy. In addition, quantitative studies will be conducted: the first will estimate the cost of the diagnostic odyssey that could potentially be avoidable with first-line WGS_T_ in all patients previously investigated in the DEFIDIAG study; the second will estimate changes in follow-up of the patients in the year after the return of the WGS_T_ analysis compared to the period before inclusion. Finally, through semi-directive interviews, we will explore the expectations of 60 parents regarding genomic analyses.

**Discussion:** Humanities and social sciences studies can be used to demonstrate the efficiency of WGS and assess the value that families associate with sequencing. These studies are thus expected to clarify trade-offs and to help optimize the implementation of genomic sequencing in France.

**Ethics Statement:** The protocol was approved by the Ethics Committee Sud Méditerranée I (June 2019)—identification number: 2018-A00680-55 and the French data privacy commission (CNIL, authorization 919361).

**Clinical Trial Registration**: (ClinicalTrials.gov), identifier (NCT04154891).

## Introduction

Genomics in medicine is profoundly modifying our understanding and our clinical practices. To ensure that access to these new technologies is equitably distributed throughout the country, France launched in 2016 a national plan for genomic medicine (Aviesan, 2025; PFMG 2025), similarly to what had already been implemented in other countries (Collins et al., 2003; Zhao et al., 2004; Peplow, 2016; Aviesan, 2025). This national plan aims to change the way patients are diagnosed, followed-up, and treated by 2025 in various medical specialties such as cancer and rare diseases, and to extend this to common diseases (Zhao et al., 2004). By setting up routine genome sequencing, the ambition is to enable more personalized diagnosis and therapeutic management of patients.

Rare diseases are considered to be at the forefront for the implementation of next generation sequencing (NGS) in France as part of the developing genomic strategies. Among rare diseases, intellectual deficiency (ID) is the most common reason for referral in genetic centers. This neurodevelopmental disorder is characterized by intellectual quotients (IQ) under 70 before the age of 18. It affects between 1 and 3% of the general population, with around 15 per 1,000 persons with mild ID and around 3 per 1000 with severe ID (Buntinx et al., 2016). ID is extremely genetically heterogeneous, which makes it difficult to obtain a genetic diagnosis. The combination of these characteristics has made ID a major public health challenge, and there is an urgent need to improve the rate of diagnosis for individuals with ID in order to offer them optimal care.

The emergence of NGS is a real technological breakthrough for the molecular diagnosis of ID. It was used to develop gene panel sequencing (GPS), in which a selection of genes is captured and sequenced, whole exome sequencing (WES), and more recently whole genome sequencing (WGS). In France, the sequencing strategy for rare diseases is still under debate, and studies are needed to define the diagnostic yield of the different NGS techniques as well as replacement of chromosomal microarray analysis (CMA) by WGS for instance. The evaluation of the efficiency of GPS and WES compared to the strategy using Fragile-X (Fra-X) testing, CMA and a gene panel of 44 selected ID genes, which has been historically used in France, is ongoing through an economic research program funded by the French Ministry of Health. But WGS constitutes another apparent opportunity for patients with ID. Its diagnostic yield is estimated to be 60–68% (Van Nimwegen, 2017) compared with 43% using WES (Gilissen et al., 2014) and up to only 32% using GPS (Redin et al., 2014; Chérot et al., 2018).

To determine the optimal conditions for the implementation and the generalization of WGS in clinical practice in a context of limited resources, decision-makers need to be provided with complete and comprehensive information. In this context, one of the four pilot studies of the PFMG 2025, the DEFIDIAG study, was dedicated specifically to ID. Beyond the technical challenge of improving the diagnostic yield, this study represents an opportunity to explore other humanities and social issues, which concern the patients and their families, the clinicians, the payers and decision-makers, and the health care system as a whole. Studies have been already been published on the complete cost and/or efficiency of WGS (Jegathisawaran et al., 2020; Yuen et al., 2018; Tslipova et al., 2017; Van Nimwegen, 2017; Ontario Health, 2020; Schwartze et al., 2020). Some recent studies also focused on the diagnostic costs avoided by the use of first-line genetic WES (Monroe et al., 2016; Stark et al., 2017; Vissers et al., 2017) and more rarely WGS (Ontario Health, 2020). But heterogeneous clinical presentation, sample sizes, and differences in methodologies used make it difficult to compare these results and to generalize to other settings. Finally, authors already explored the perception that families have of diagnostic results (Foster et al., 2009; Kohler et al., 2017; Mollison et al., 2020), but to the best of our knowledge, no study has explored their experience of the entire process of care (from the prescription of genetic analysis until the disclosure of results and a post-result period). A longitudinal investigation may help to better understand their needs and expectations as well as their perception of the utility of the results for themselves and for their relatives.

In this context, this article aims to present the humanities and social sciences (HSS) dimensions of WGS in the French context based on the population of the DEFIDIAG project. Four dimensions will be explored: 1) efficiency of WGS, 2) impact of WGS on cost savings, 3) impact of WGS on the medical, medico-social, rehabilitative and psychological follow-up of patients presenting ID and 4) investigation of the experience of the parents concerning the health care pathway as a whole.

## Methods and Analyses

The HSS study is part of the DEFIDIAG project, which is a prospective multicenter diagnostic study (Binquet et al., 2022).

### Summary of the DEFIDIAG Study

The main goal of the DEFIDIAG project (NCT04154891) is to compare the percentage of ID causal diagnoses obtained using 2 different strategies: WGS using a trio strategy (WGS_T_) and the reference strategy [*i.e.*, use of the French guidelines based on the ANPGM (National Association of Molecular Genetics Practitioners)] applied blindly to consecutive patients with no obvious diagnosis. The population is composed of patients between 0 and 5 years meeting stringent criteria (severe delayed development in terms of motor skills, language, and/or sociability) or patients older than 6 years whatever the ID severity (but with proven ID by *ad hoc* neuropsychological testing) and the associated manifestations, and without any obvious diagnosis identified during a genetic consultation.

A total of 1,275 patients will be then included in one of the 14 participating clinical centers, with 50% of patients coming for their first consultation (patients never explored) and the other 50% having had a previous genetic exploration. Each included patient will be their own control; they will benefit from the two main strategies in parallel. WGS_T_ and the reference strategy will be compared in 7 subgroups: 3 subgroups defined according to age (<2 years old/2–5 years/>5 years), 4 subgroups of patients defined according the severity of ID, and/or with associated manifestations (ID associated with major non-cerebral manifestation, moderate to severe ID, mild ID associated with another sign, ID associated with epilepsy). The diagnostic yield of WGS using a simplex strategy (WGS_S_) will be also investigated in parallel to the two main strategies, but only in a randomized subgroup of the overall population consulting a geneticist for the first time.

### Objectives of the HSS Study

The HSS study consists of four parts, which have been designed in conjunction with the DEFIDIAG study:

- a *cost-effectiveness analysis* that will be undertaken in the population consulting a geneticist for the first time.
- a study aiming to estimate *the cost of the diagnostic odyssey* which could have potentially been avoided with a first-line WGS_T_ in previously investigated patients.
- a study whose goal will be to estimate the *frequency and nature of changes in patient follow-up* in the first year after the WGS_T_ analyses compared to the period before inclusion for both previously investigated patients and the population consulting for the first time.
- a *qualitative study* which will explore parent expectations regarding the genomic analyses, how they feel about the results and how they perceive the future.

The whole DEDIDIAG study, including the HSS analyses, has been approved by ad hoc ethics committee (Identification number 2018-A00680-55). The inclusion of patients is ongoing.

### Methods of the HSS Studies

#### Efficiency Study

##### Compared Strategies

**FIGURE 1:**
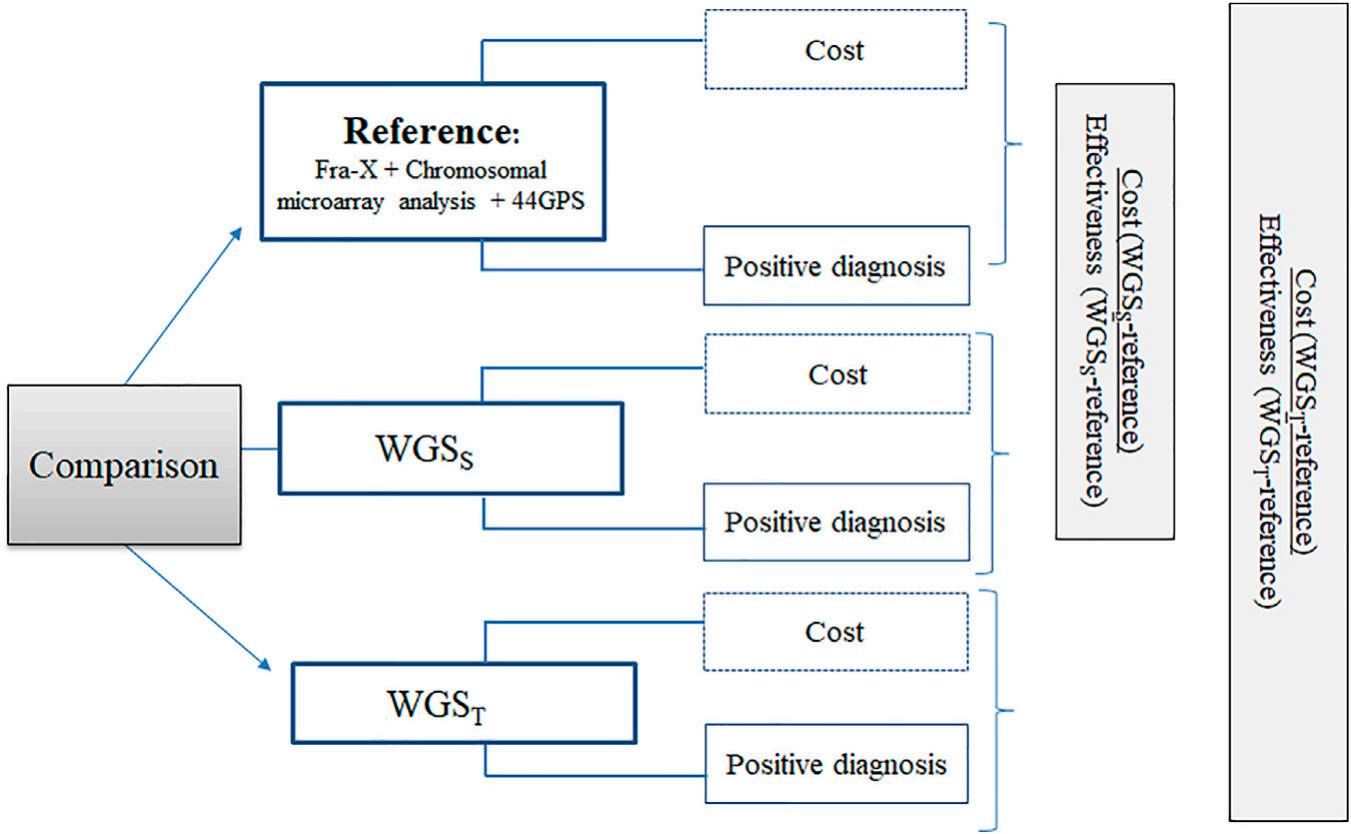
Design of the efficiency study: This figure illustrates the design of the cost-effectiveness study. Three strategies will be compared: the solo Whole Genome Sequencing strategy (WGS_S_), the trio Whole Genome Sequencing strategy (WGS_T_) and the reference strategy. Comparisons will be made simultaneously in terms of cost and effectiveness (positive diagnosis).

The aim of this efficiency study is to compare the following three strategies in terms of cost and effectiveness for the causal diagnosis of ID in the first investigated population: the French core minimal reference strategy combining Fra-X testing, CMA, and a panel of genes commonly known to be involved in ID (44 GPS), WGS_T_ and WGS_S_ (Figure 1).

##### Sample Size

Overall 9 comparisons had to be performed in the main project given the 7 subgroups to account for and the comparison between WGS_T_ and WGS_S_. Thus, the alpha risk was set to 0,00278 and the target power to 80%. A difference of 7% was expected between WGS_T_ and WGS_S_ (66–68% as reported by Gilissen et al., 2014 for WGS_T_ and 60% by Lionel et al., 2017 for WGS_S_) and less than 0.1% of diagnosis identified by WGS_S_ and not by WGS_T_. Assuming these assumptions, the sample size required was 189 patients. We planned to include 7 additional patients (3.5%) to account for unusable samples or other technical problems.

##### Effectiveness

The effectiveness criterion used in the cost-effectiveness analysis will be the primary study end point of the DEFIDIAG study, *i.e.*, the identification of a causal diagnosis of ID defined as the identification of one class 4 (likely pathogenic) or 5 (pathogenic) variant that explains the symptoms presented by the patient.

##### Identification and Measurements of Data for the Estimation of Costs

The planned time frame for the cost-effectiveness analysis is 12 months, which is the estimated maximum time required to perform from the WGS_T_ and WGS_S_ analyses and interpret the data, and to return the results to the patient and their parents.

The economic evaluation will be conducted from the point of view of the “collectivity,” as recommended by the French National Health Authority (HAS), meaning that all the stakeholders involved in the decision are considered. In this perspective, the production costs of strategies should be identified, measured and valued independently of their current or envisaged sources of funding. Thinking in terms of production costs makes it possible to have an in-depth analysis of the resources used and allows the stakeholders to think about their participation in terms of funding, and to determine or adjust tariffs.

Only direct medical costs will be considered. They will include: (i) consultation with the clinical geneticist in the recruiting centers, as well as exams preceding the genetic analysis and inclusion in the DEFIDIAG project (brain magnetic resonance imaging (MRI) and neuropsychological assessment carried out according to the French recommendations); (ii) first blood sample; (iii) Fra-X testing, CMA, 44GPS, WGS_T_/WGS_S_ and (iiii) possible complementary (imaging) and confirmatory exams (such as Sanger and qPCR: quantitative Polymerase Chain Reaction). The first four categories of costs will be recorded for each patient. To identify the complementary examinations, investigator physicians will be required to indicate the additional examinations/visits that they would consider prescribing for each patient in the e-Case Report Form (e-CRF) at the time of inclusion. They must complete the form for each of the three strategies independently of each other. Physicians will also be asked to indicate which confirmatory techniques they judge useful to perform for each strategy before unblinding and to record this information in the e-CRF during the multi-disciplinary meeting (MDM). The economic analysis will also consider the fact that these analyses could have been performed or not. If they are performed, the date will be collected. The use of a declarative method to identify the examinations is justified by the blinded design of the study.

##### Monetary Valuation of Ressources

The transport costs for the blood samples will be valued on the basis of invoices. Existing tariffs will be used for valuation of all other medical, biological and technical exams. The use of tariffs is considered acceptable by the HAS because they are considered as the counterpart of the resources consumed for carrying out these analyses. Otherwise, the microcosting method will be used to estimate the production costs of WGS_T_ and WGS_S_ (Frick, 2009). The cost will be obtained by considering the volumes of each mobilized resource (labor, disposable and reagents, material, equipment, etc.) and their associated monetary value (mean gross wages, purchase price of consumables, software, and equipment, etc.). The microcosting method will require the development of grids for collecting the resources used during the pre-analytical (DNA extraction and quality control), analytical (sample bank preparation, production, quality control and primary bioinformatic analysis including data-storage, bioinformatic and the post-analytical (interpretation by biologists, multidisciplinary meeting discussions and disclosure of results to the parents and patients) steps. The cost of the re-analysis of variants of unknown significance will not be taken into consideration because it goes beyond the 12-months time horizon of the analysis.

#### Cost of the Diagnostic Odyssey

**FIGURE 2:**
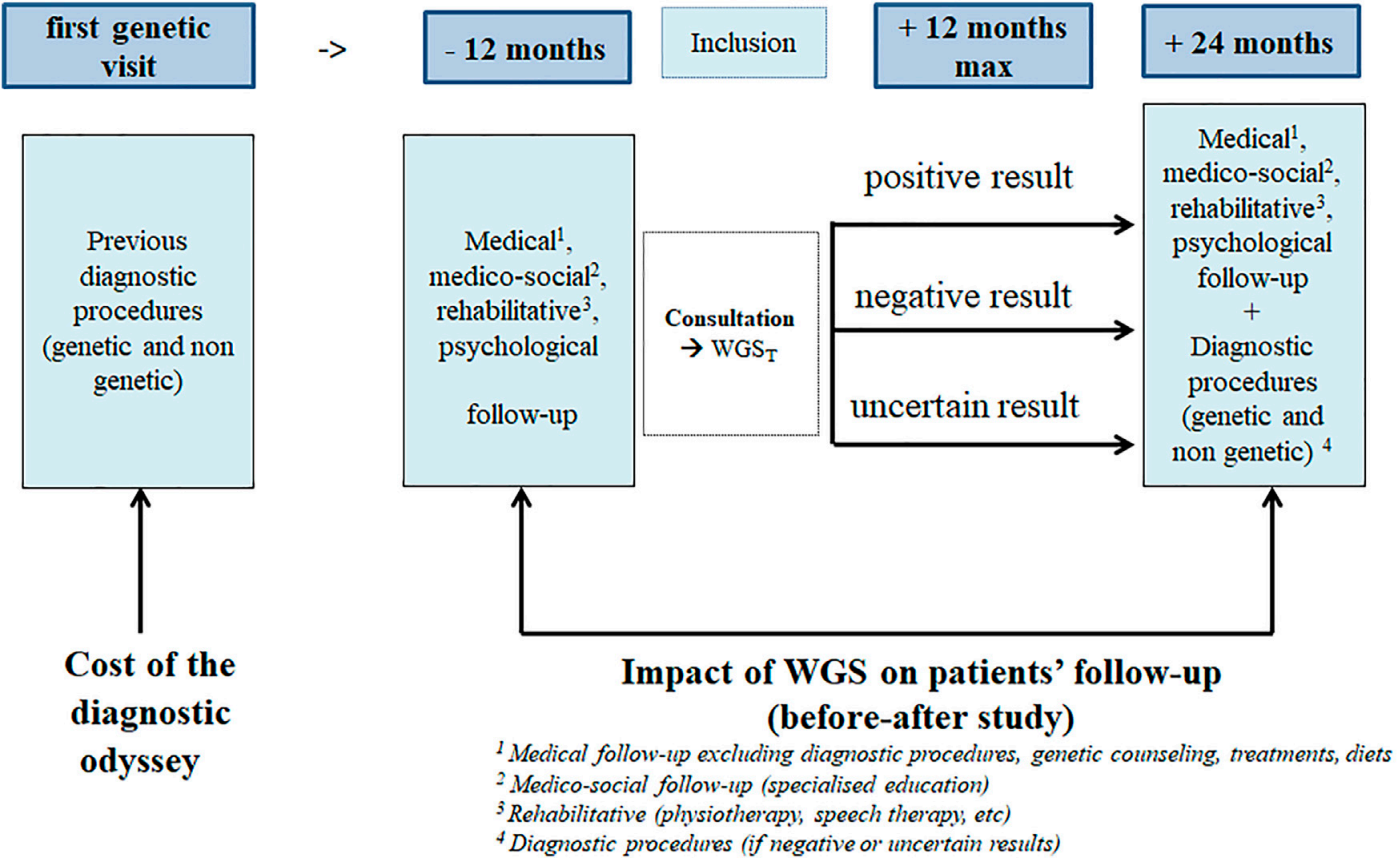
Design of the impact of genome sequencing on the diagnostic odyssey and follow-up: This figure illustrates the design of the before-after study aiming at assessing the impact of WGS on patients’ follow-up (12 months after the reporting of WGS results) compared to the period of 1 year preceding the inclusion.

The cost-effectiveness analysis will be completed by the estimation of the costs related to the iterative search for a diagnosis in the *previously investigated* population. The search could potentially include genetic investigations, biological tests, imaging procedures, specialized consultations, and/or hospitalization (Figure 2). The collection of previous examinations will be based on the investigation of the patient’s medical file and the consultation including the medical geneticist, the patient and their parents. In case of discordance between the data sources, the interviews will be considered the definitive source to be used for the analysis. All previous examinations will be valued with the current tariffs. In case of hospitalisation, the cost of daily hospital stays will be estimated from the Diagnosis Related Groups (DRG) associated with each inpatient stay. They will be identified using the patient’s main diagnosis, the medical procedures performed during the hospitalization, and the status of the health care centre (public, private). The cost of each DRG will be then estimated using a National Cost Survey Sample, named “*Echelle Nationale des Coûts*” (ENC), which provides costs data based on a sample of centres in France. The identification of DRGs and their monetary valuation will be performed by each health care centre included in the DEFIDIAG project.

#### Impact of WGS on Patient Follow-Up

A before-after study is planned aiming to assess the frequency and nature of changes in medical follow-up (treatments, diets, medical supervision, genetic counseling to the patients and relatives), as well as medico-social (education, type of institution attended), rehabilitative (physiotherapy, speech therapy, etc.), and psychological follow-up in the first year after the reporting of WGS_T_ analyses (whether the results are positive, negative or uncertain), and to compare the findings to a period of 1 year maximum preceding the inclusion (Figure 2). In the “before” period, the consultation between the medical geneticist, the patient and their parents will allow us to collect the data which could be completed by medical files. In the “after” period, parents will be asked to fill in a diary. Quarterly, a clinical technician will call the parents to fill the e-CFR with the recorded data.

#### Qualitative Study

A qualitative study will be based on semi-directive sociological and psychological interviews with the parents of the patients at three different times: at inclusion, a few days after the WGS results are disclosed and 1 year after the results are disclosed. At inclusion, the family history, the representation of genetics, and the expectations regarding the genomic analysis will be explored. We will also explore how they anticipate the waiting time for the results (how do they think they will react and what support they would like at this time). After the disclosure of results, the objective is to assess the reaction of the family and how they anticipate the future, and at 1 year, the possible changes that the families have experienced, how they feel about the genomic analysis, and their expectations about the future.

Parents will be included in two main centres participating in the DEFIDIAG study: Dijon University Hospital and Pitié-Salpétrière University Hospital (Paris). The choice of these centers is justified by the need to have a diversity of parent profiles in terms of both socio-economic and cultural background. The qualitative study will be performed in a sub-sample of the whole population of the efficacy study. We hypothesized that 60 interviews (30 in sociology and 30 in psychology) will be sufficient to achieve data saturation in each approach (Hennink et al., 2017) since the qualitative template has been built by HSS researchers and that questions will be similar whatever their HSS background (Table 1). Inclusions of patients coming for a first consultation have to be as consecutive as possible to meet the standards of a diagnostic study, therefore guaranteeing the heterogeneity of the demographic, and also cultural and socio-economic profiles of parents. If possible, the parents will be stratified in subgroups (first investigation population vs. previously investigated population) and according to the clinical profile of the patient (mild, moderate or severe/profound ID).

**TABLE 1:** Template of the qualitative study.

Interview 1: After the inclusion in the DEFIDIAG study		
Trajectory of care	Experience of the care pathway	Can you tell me about your child’s care pathway?
		-Support
		-Care, type of relationship with professionals
		-Organization for daily life
	Representation of difficulties (historicity)	What were the difficulties encountered?
		-Access to knowledge
		-Information flow
		-Feelings at diagnosis/diagnosis delay
Positioning in relation to genetic research	Représentation de la génétique	What are your expectations regarding genetic research?
		-How long have you been searching for the reasons for your child’s disability?
		*Reminder: here there will be a distinction between de novo versus parents who have been searching for years and differences in perception*
		-Fears and hopes possibly in the process of recovery
	Representation of genetics	- Could you tell me why you agreed to participate in the DEFIDIAG study?
		-What information did you receive about the study?
	Anticipation genetic results	- How do you feel about waiting for the results?
		-How do you think you will react?
		-What support would you like at this time?
	Secondary data	-What were the reasons for your decision to seek secondary data?
		→Do you have any particular expectations?
End		-Could you tell me three words that describe how you feel now?
**Interview 2: After the results’ disclosure**		
Introductory question: Which results did you receive?		
The experience of the announcement of the results	Subjective dimension	Could you tell me how you felt when you got your results?
		- personal reactions
		- reactions of those around you
		- reactions to the people around you
	Context of the announcement	-Could you tell me how the results were announced to you?
Impact of the announcement of the results	Reception and appropriation of results	-What did you retain from the information given?
		-Did it raise any questions?
		-With whom did you discuss it?
Perception of the future	Expected changes	-Do you think these results will change things?
		-Which ones?
		-In the short/medium/long term
		- Do you think it will change the relationship you have with your child?
	Feeling	-Do these results lead to changes in perspective?
		-For you
		-For your child
		-Did you have any knowledge of the diseases mentioned?
		-Representations
		-Did you share these results with other family members? How did they react to this information?
Primary and secondary data		What if you had to do it again?
End		-Could you tell me three words that describe how you feel now?
**Interview 3: 1 year after the disclosure of the results**		
Changes since the announcement of the results	Organization of daily life	-Have there been any changes since the results?
		→concretely?
	Subjective dimension	-How do you feel since the results?
		-How often do you think about the results?
		-How have you experienced the period after the results?
		-Are there any changes in the way you think about the disease/treatment/follow-up?
		-Are there any changes in your relationships with your family and friends?
Evolution of the perception of genetics		-What do you think of the care provided by the genetics team?
		-Do you want to know the full results of the study?
If secondary data disclosure	Appropriation of results and feelings	-What did you learn about the secondary data
		-Have you discussed the secondary data with others?
		-Did the results change anything in your daily life?
		-Have you had/are you considering further tests?
		What if you had to do it again?
End	Perception of the future	-How do you see the future?
		-What kind of support would you like for the future?
		-Could you tell me three words that describe how you feel now?

Data concerning the family situation, the number of children (with and without disability), and the deprivation level (working status and education level) will be recorded at inclusion. Their phone number and current address will also be collected in order to provide this information to the sociologist and the psychologist, who will contact the participants to define a date and a place for the interview. The interview can take place at the health care center, at home or in a neutral place at the convenience of the participants. A phone call or a videoconference will be also possible. The parents can choose whether they prefer to be interviewed together or not.

### Analysis

#### Efficiency Analysis

The efficiency criterion will be based on the estimation of an incremental cost-effectiveness ratio (ICER), expressed in terms of cost per additional positive diagnosis. The results will not be discounted given the time horizon. A deterministic analysis will be used to consider evolutions in the technological field, such as the use of different generations of sequencing machines and the automatization of some steps which could modify the relative part of labor in the cost estimation. Another analysis involves the completion or non-completion of the complementary and confirmatory examinations that will be carried out at the time of the inclusion (the main analysis will consider only the prescribed acts that were actually performed). A probabilistic analysis based on a non-parametric bootstrap analysis will be also performed in order to manage the uncertainty associated with sampling and to estimate the 95% confidence interval of the ICERs (Briggs and Alastair, 1999). Given the number of strategies compared (> 2), we will estimate the net monetary benefit (NMB) associated with each of the strategies of the study. The NMB represents the value of an intervention in monetary terms when a willingness to pay threshold (ʎ) for a unit of benefit (E) is known (York Health Economics Consortium, 2016). A positive NMB indicates that the intervention is cost-effective compared with the alternative at the given willingness-to-pay threshold. The NMB is calculated for each selected value of ʎ, making it possible to plot the acceptability curves for each strategy. This analysis will enable decision-makers to further view the results of this evaluation in terms of their budgetary reality.

#### Cost of the Diagnostic Odyssey

The costs of the diagnostic odyssey will be described only in the population of previously investigated patients. The results will be expressed as means and standard deviations in case of normal distribution, and as medians with interquartile ranges otherwise. A subgroup analysis may be performed to specifically target ID characteristics. Mean costs will be then compared by an analysis of variance or by a Kruskall-Wallis test according to the conditions of application. A *p*-value below 0.05 is considered as significant.

#### Impact of WGS on Patient Follow-Up

The frequency at which changes are made to patient follow-up between the period prior to inclusion and the period following the results will be calculated with the 95% CI. A global analysis will be then performed, whatever the result of WGS_T_ (positive, negative, non-conclusive). Sub analyses will be then conducted according to the result of WGS_T_. We will also provide a description of the new medical diagnostic procedures performed among patients whose results are negative or uncertain with the WGS.

#### Qualitative Study

The analysis of the interviews will be based on the following steps: open codification of transcribed interviews to identify as many topics as possible in the initial corpus; categorization of codified elements; careful re-reading of the corpus as a whole with the aim of clearly defining each category; linking categories; writing more detailed memos and designing explanatory diagrams; integration of the previous steps in order to identify the main points of the phenomenon; and theorization: the meticulous and exhaustive construction of the “multidimensionality” and the “multicausality” of the phenomenon of the relationships between needs, expectations, hopes, suffering, and the results of genetic analysis. For the psychological aspect, the interviews will be analysed using the general inductive method (David and Thomas, 2006), which uses the first three steps described above.

## Discussion

This part of the DEFIDIAG project illustrates the complementarity of the various HSS methodologies and their ability to extend beyond the primary goal of the initial study. Our ambition is to consider the economic, medical, sociological, and psychological dimensions of genomics in order to provide French decision-makers with in-depth information about the advantages and constraints of WGS. In the economic field, one recent study demonstrated that WGS was about US$ 1,000 less expensive than standard testing (which included CMA, Fra-X, targeted single-gene tests and GPS) and was more than two-fold effective (in number of molecular diagnoses) (Ontario Health, 2020). Such data is lacking in the field of ID in France. Another goal is to provide WGS complete costs. Schwarze et al. provided the first data in the United Kingdom in the field of a rare disease trio case based on the microcosting method. The cost was estimated to be £ 7,050 (US $ 9,330) per genome (Schwartze et al., 2020). Other results which have already been published presented various estimations from € 1,421 (US $ 1,602) (Van Nimwegen, 2016) to CAN $ 6,435 (US $ 4,975) (Jegathisawaran et al., 2020), mainly explained by differences in terms of methodology. Results are also dependent from local organizations. In France, this kind of data is essential to obtain to contribute to the determination of tariffs to be reimbursed by the national health insurance. This result will be then used in our cost-effectiveness analysis. These results could also be used in a decision-analysis model to consider the improvement in diagnostic performance with WGS.

One potential limit relative to this planned economic evaluation as part of the DEFIDIAG project is that effectiveness will be expressed in terms of the number of positive diagnoses. Our choice is justified by the need to remain consistent with the primary goal of the study. Cost-utility analyses are commonly used alongside economic evaluations. In these studies, effectiveness is expressed in terms of QALYs (Quality-adjusted Life-years), a bidimensional criterion based on the quantity of life lived weighted by an “utility” score which represents the satisfaction that patients attribute to a health state. One QALY equals 1 year of life in perfect health. But the first challenge of ID is to explain the disease by providing the parents with a causal diagnosis and decreasing the negative impact or burden of the impairment (Adithyan et al., 2017). In the state of the art, the questionnaires used today to assess utility (*e.g.,* EQ-5D™) are not totally adapted to ID. Moreover, economic evaluations can not yet combine the point of view of the parents and the patients. However, it is important to consider how genomic testing impacts families. As an alternative, we decided to obtain a longitudinal comprehensive view of the experience of the families during the genetic and care pathway of their children in the first year after the disclosure of the results. These data are fundamental to adjust, if necessary, the support provided to these families. Previously published studies on the perception that families have of the diagnostic results generally focused on the concept of “personal utility” (Foster et al., 2009; Kohler et al., 2017; Mollison et al., 2020). We choose not to base the interview questions on a specific concept. Concepts and models will be identified from the interviews using an inductive approach. The collected parameters, such as the age of the parents, their socio-economic level as well as the demographic and clinical profile of their child will be useful for the interpretation phase. Religious and spiritual beliefs and practices will not be collected, but the interviews make it possible to consider these aspects given the fact that the question of the value associated with genetics will be explored. Given the fact that eligible patients have to be as consecutive as possible to meet the standards of a diagnostic study, heterogeneity of the sampling of parents for the interviews will be guaranteed. Finally, the HSS DEFIDIAG study planned to assess the changes in the follow-up of the patients 1 year after the results. To our knowledge, the evaluation of a similar objective was conducted only in one monocentric retrospective study. It compared the pre-WES to the post-WES costs among patients with ID, but only from a diagnostic point of view; neither the impact on treatments nor the impact for patients presenting negative result was considered (Vrijenhoek et al., 2018).

To conclude, decision-makers need to be given a clear demonstration of the efficiency of WGS, to be informed about how WGS will affect the medical care pathway, and to fully understand the fear and expectations of families, all of these factors are among the conditions required for its successful generalisation.

## Trial Status

Recruiting is ongoing (1 224/1 275 patients included as of 03/01/2022).

## Full List of Co-Investigators of the DEFIDIAG Study Group

**Centre National de Recherche en Génomique Humaine et al.**, Centre National de Recherche en Génomique Humaine: MEYER Vincent; CHU d’Angers: BONNEAU Dominique, BARTH Magalie, TESSARECH Marine, ZIEGLER Alban; CHU de Bordeaux: GOIZET Cyril, LACOMBE Didier, LEGENDRE Marine, MARGOT Henri, MICHAUD Vincent, NAUDION Sophie, ROORYCK THAMBO Caroline; **Dijon-Bourgogne and Inserm UMR1231- Equipe**, CHU Dijon-Bourgogne & Inserm UMR1231- Equipe GAD: BOURNEZ Marie, BRUEL Ange-Line, COLIN Estelle, DELANNE Julian, DENOMME-PICHON Anne-Sophie, GARDE Aurore, MOUTTON Sébastien, NAMBOT Sophie, PHILIPPE Christophe, SAFRAOU Hana, SORLIN Arthur, THAUVIN Christel, TRAN-MAU-THEM Fréderic, VITOBELLO Antonio; **Grenoble-Alpes et al.**, CHU Grenoble-Alpes: DIETRICH Klaus, DURAND Chantal, MAREY Isabelle, N’GUYEN-MOREL Marie-Ange, THEVENON Julien; **CHU de Lille et al.**, CHU de Lille: BOUTE Odile, CAUMES Roseline, COLSON Cindy, DIEUX Anne, GHOUMID Jamal, MARSILI Luisa, PETIT Florence, VANLERBERGHE Clémence, VINCENT-DELORME Catherine; **Hospices Civils de Lyon et al.**, Hospices Civils de Lyon: ARMAND Thibaud, CHATRON Nicolas, CURIE Aurore, DES PORTES Vincent, EDERY Patrick, HAYE Damien, LABALME Audrey, LESCA Gaëtan, MONIN Pauline, PONS Linda, PUTOUX Audrey, ROSSI Massimiliano, ROUGEOT Christelle, TILL Marianne; **CHU de Montpellier et al.**, CHU de Montpellier: BLANCHET Patricia, COUBES Christine, DEILLER Caroline, GENEVIEVE David, PINSON Lucile, WELLS Constance, WILLEMS Marjolaine; **CHU de Nantes et al.**, CHU de Nantes: ISIDOR Bertrand, MERCIER Sandra, NIZON Mathilde, VINCENT Marie; **Hôpital Necker-Enfants Malades**, Hôpital Necker-Enfants Malades (AP-HP): AMIEL Jeanne, BARCIA Giulia, BAUJAT Geneviève, CORMIER Valérie, GUIMIER Anne, HADJ ABDALLAH Hamza, MALAN Valérie, MARLIN Sandrine, MARZIN Pauline, MICHOT Caroline, ORMIERES Clothilde, RIO Marlène, ROMANA Serge; **Groupe Hospitalier Pitié-Salpêtrière et al.**, Groupe Hospitalier Pitié-Salpêtrière (AP-HP): AFENJAR Alexandra, BURGLEN Lydie, CHARLES Perrine, COURTIN Thomas, HEIDE Solveig, KEREN Boris, LEHALLE Daphné, MIGNOT Cyril, MOUTHON Linda, WHALEN Sandra; **CHU de Rennes et al.**, CHU de Rennes: FRADIN Mélanie, JEAN-MARCAIS Nolwenn, LAVILLAUREIX Alinoë, MOREL Godelieve, PASQUIER Laurent, QUELIN Chloé, RIOU Audrey, UGOLIN Mélissa; **CHU de Rouen: BREHIN Anne-Claire et al.**, CHU de Rouen: BREHIN Anne-Claire, CASSINARI Kévin, CHAMBON Pascal, GOLDENBERG Alice, GUERROT Anne-Marie, JOLY-HELAS Géraldine, LECOQUIERRE François, LEMEUR Nathalie, NICOLAS Gaël, SAUGIER-VEBER Pascale, VERA Gabriella; Hôpitaux Universitaires de Strasbourg: EL CHEHADEH Salima, CALMELS Nadège, HAUSHALTER Virginie, MAILLARD Pierre-Yves, MULLER Jean, PHILIPPE Anaïs, PITON Amélie, SCHAEFER Elise, SCHEIDECKER Sophie, SCHLUTH-BOLARD Caroline; Hôpital de la Timone (Hôpitaux Universitaires de Marseille): BUSA Tiffany, PHILIP-SARLES Nicole, RICCARDI Florence, SIGAUDY Sabine; Institut Imagine: NITSCHKE Patrick.

## Data Availability

The original contributions presented in the study are included in the article/Supplementary Material, further inquiries can be directed to the corresponding author.

## Abbreviations

HAS, French national health authority; ANPGM, national association of molecular genetics practitioners; CMA, chromosomal microarray analysis; DNA, deoxyribonucleic acid; DRG, diagnosis related groups; ENC, echelle nationale des coûts; WES, whole exome sequencing; e-CRF, e-case report form; GPS, gene panel sequencing; 44GPS, 44 gene panel strategy; HSS, humanities and social sciences; ICER, incremental cost-effectiveness ratio; ID, intellectual deficiency; MRI, magnetic resonance imaging; NGS, next generation sequencing; NMB, net monetary benefit; PFMG 2025, plan france médecine génomique 2025; WGS, whole genome sequencing; WGS_T_, trio whole genome sequencing; WGS_S_, solo whole genome sequencing.
